# Allelic Variation and Distribution of the Major Maturity Genes in Different Soybean Collections

**DOI:** 10.3389/fpls.2018.01286

**Published:** 2018-09-04

**Authors:** Jegor Miladinović, Marina Ćeran, Vuk Đorđević, Svetlana Balešević-Tubić, Kristina Petrović, Vojin Đukić, Dragana Miladinović

**Affiliations:** ^1^Soybean Department, Institute of Field and Vegetable Crops, Novi Sad, Serbia; ^2^Industrial Crops Department, Institute of Field and Vegetable Crops, Novi Sad, Serbia

**Keywords:** photoperiodism, *E* genes, soybean, maturity, allelic variation, SNP

## Abstract

Soybean time of flowering and maturity are genetically controlled by *E* genes. Different allelic combinations of these genes determine soybean adaptation to a specific latitude. The paper describes the first attempt to assess adaptation of soybean genotypes developed and realized at Institute of Field and Vegetable Crops, Novi Sad, Serbia [Novi Sad (NS) varieties and breeding lines] based on *E* gene variation, as well as to comparatively assess *E* gene variation in North-American (NA), Chinese, and European genotypes, as most of the studies published so far deal with North-American and Chinese cultivars and breeding material. Allelic variation and distribution of the major maturity genes (*E1, E2, E3*, and *E4*) has been determined in 445 genotypes from soybean collections of NA ancestral lines, Chinese germplasm, and European varieties, as well as NS varieties and breeding lines. The study showed that allelic combinations of *E1*–*E4* genes significantly determined the adaptation of varieties to different geographical regions, although they have different impacts on maturity. In general, each collection had one major *E* genotype haplogroup, comprising over 50% of the lines. The exceptions were European varieties that had two predominant haplogroups and NA ancestral lines distributed almost evenly among several haplogroups. As *e1-as/e2/E3/E4* was the most common genotype in NS population, present in the best-performing genotypes in terms of yield, this specific allele combination was proposed as the optimal combination for the environments of Central-Eastern Europe.

## Introduction

Photoperiodism is reaction of a plant to the length of day and night. The discovery of photoperiodism in soybean and tobacco (Garner and Allard, 1930), and subsequently in numerous other plant species, paved the way for investigation of mechanisms which control seasonal rhythm in plants, such as flowering, growth and reproduction (Putterill et al., 2009). Besides plant growth, photoperiod also affects other aspects of plant development, including changes of developmental phases and overall length of vegetation period. Knowledge of photoperiodic response of cultivated crops is one of the vital aspects of modern-day plant production, as shifting seasonal timing of reproduction became a major goal of plant breeding programs in their effort to produce novel varieties better adapted to local environments and variable climate conditions. This also stands for soybean [*Glycine max* (L.) Merr.] since it is a photoperiod-sensitive short-day plant, i.e., soybean transition from vegetative to reproductive stage directly depends on the length of day (Miladinović and Đorđević, 2011).

## Soybean and Photoperiodicity

Estimation of plant phenological stage of development, as a function of specific environment, is the key factor in any attempt of modifying plant growth, adaptation, or productivity (Jones and Laing, 1978; Davidson and Campbell, 1983; Hodges and French, 1985; Wang et al., 1987; Colson et al., 1995; Miladinović and Đorđević, 2011). Soybean phenology is difficult to predict, as it depends on photoperiod (Garner and Allard, 1930; Seddigh et al., 1989), temperature (Egli and Wardlaw, 1980; Board and Hall, 1984), and amount of plant-available water (Meckel et al., 1984; Miladinović et al., 2006).

Dependence of soybean on the length of the day resulted in its geographic distribution into 13 maturity group (MG) zones, from MG000 comprised of varieties which can thrive in higher geographic latitudes, to MGX which includes varieties grown in lower latitudes (Hartwig, 1973). By definition, MGs are the result of classification of soybeans based on their growth and development. The difference between MGs in a certain area are caused by photoperiodic requirements of a variety, while the difference in maturity date between two adjacent MGs ranges from 10 to 18 days. In soybeans, the critical photoperiod, or the definite day length light period above or below which the plant never blooms, decreases progressively from higher to lower geographic latitudes. Photoperiodic requirements thus reduce soybean growing area to a narrow latitude range of around 200 km (Scott and Aldrich, 1983). If grown in higher geographic latitudes compared to its optimal growing area, a soybean variety will flower and mature later, while a variety grown in lower geographic latitudes will flower and mature earlier, resulting in lower vegetative mass and lower yields. Hence, there is an optimal MG for each soybean growing region (Miladinović and Đorđević, 2011).

## Soybean and *E* Genes

Time of flowering and maturity in soybean are genetically controlled by *E* genes that have different roles in maturity and photoperiod sensitivity. Their allelic combinations contribute to fine adaptation of genotype to certain latitude and climate conditions.

Up to date, 11 major loci (*E1*–*E10* and *J*) affecting time of flowering and maturity have been identified in soybean (Samanfar et al., 2017). The dominant allele of *E* genes usually confers later flowering and later maturity, except for *E6, E9*, and *J* (Ray et al., 1995; Bonato and Vello, 1999; Kong et al., 2014), while photoperiod sensitivity decreases with numbers of recessive alleles. Owen (1927) described the effect of *E1* gene in soybean in his inheritance studies, while Bernard (1971) described two major genes (*E1* and *E2*) which control flowering time and maturity. Buzzell (1971) and Kilen and Hartwig (1971) investigated soybean flowering response to fluorescent daylength conditions and concluded that *E3* gene determines late flowering, while *e3* causes insensitivity to fluorescent light. Buzzell and Voldeng (1980) studied inheritance of insensitivity to extended daylength and found that *E4* allele confers late flowering and sensitivity to extended daylength, while *e4* allele influences early flowering and insensitivity to extended daylength. McBlain and Bernard (1987) described *E5* gene and its genetic effect similar to *E2*. However, in the study conducted in order to map *E5* locus, the results obtained from different F2 mapping populations expected to segregate for *E5* were not consistent, and no candidate QTL was found, indicating that a unique *E5* gene may not exist (Dissanayaka et al., 2016). Bonato and Vello (1999) described the dominant allele of *E6* gene which confers early flowering and maturity in soybeans. Cober and Voldeng (2001) described *E7* - soybean maturity and photoperiod-sensitivity locus - and its link to *E1* and *T*. Cober et al. (2010) mapped *E8* gene, with the dominant allele resulting in later maturity and recessive allele conferring early maturity. The *E9* locus was subjected to detailed molecular analysis and gene *FT2a* was identified, with the recessive allele delaying flowering (Kong et al., 2014; Zhao et al., 2016). Samanfar et al. (2017) identified *E10*, new maturity locus in soybean, with *FT4* being the predicted functional gene underlying this locus.

Maturity genes *E1* (Xia et al., 2012), *E2* (Watanabe et al., 2011), *E3* (Watanabe et al., 2009), *E4* (Liu et al., 2008), *E9* (Zhao et al., 2016), and *J* (Yue et al., 2017) have recently been characterized at the molecular level. Tsubokura et al. (2014) estimated that maturity genes *E1*–*E4* contribute to 62–66% of the flowering time variation. Association analysis and the study of genetic architecture and networks underlying agronomical traits revealed that *E1* and *E2* loci have pleiotropic effects across the traits related to yield and seed quality, as the key nodes in the regulation of different traits (Fang et al., 2017). *E1* gene was located on chromosome 6 and identified as legume-specific transcription factor which functions as the flowering repressor with putative nuclear localization signal and B3-related domain (Xia et al., 2012). *E1* allele is functional, *e1-as* allele with missense mutation is not fully functional, while *e1-fs* with a frameshift mutation and *e1-nl* with deletion of entire *E1* gene are both non-functional (Xia et al., 2012). Mutant *e1-nl* allele was identified in MG000 European (Swedish) cultivar Fiskeby III, along with the major mutant alleles for other maturity genes (*E2* and *E3*) (Valliyodan et al., 2016). Out of the known *E* loci in soybean, *E1* gene is considered to have the largest effect on determination of flowering time under field conditions (Xia et al., 2012). For improvement of varietal adaptability, the function of *E1* gene should be reduced due to its strong impact on delaying maturity. Besides *E1*, allelic variability of other *E* genes is also important in adaptation and can provide significant genetic plasticity that could enable cultivation of soybeans in wider geographic areas (Cober et al., 1996). Located on chromosome 10, *E2* gene is an ortholog of *Arabidopsis* flowering gene GIGANTEA involved in the circadian rhythm and flowering time pathway (Watanabe et al., 2011). The dominant *E2* allele is functional, while *e2* with nonsense mutation presents the non-functional allele with an early-flowering phenotype (Watanabe et al., 2011). *E3* (GmPhyA3) and *E4* (GmPhyA2) are located on chromosomes 19 and 20, respectively, and they represent phytochrome A genes. *E3-Ha* and *E3-Mi* are functional alleles, while *e3-tr, e3-ns*, and *e3-fs* are non-functional with mutations causing truncated proteins (Watanabe et al., 2009). *E3* delays flowering under long-day conditions which ultimately affects maturity. *E4* gene has a functional allele *E4* and five non-functional alleles (*e4-SORE-1, e4-oto, e4-tsu, e4-kam*, and *e4-kes*) (Liu et al., 2008; Tsubokura et al., 2013). Being photoperiodically insensitive, varieties with non-functional alleles are mostly adapted to high latitudes, which indicates the importance of *e4* allele for high latitude adaptation. *E1* and *E2* genes have significant impact on vegetative development, while loci *E3* and *E4* might affect post-flowering reproductive development by increasing pod filling duration, and the number of nodes and pods by up-regulating the expression of growth habit gene *Dt1* (Xu et al., 2013).

Higher temperatures at reproductive stages were found to affect plant vigor and overall yield (Egli et al., 2005; Ren et al., 2009). It has also been reported that N and P concentrations in mature seeds increase with the increment of day/night temperatures (Thomas, 2001). This observation implicates that there might be some loci which show optimum activity of N uptake and transport at elevated temperatures (Patil et al., 2017). Protein content was also positively correlated with higher temperature (Wolf et al., 1982). Higher protein content was observed in late maturity group lines (MGV-MGX) compared to early maturity group lines (MG000-MGII). Since genetic variation in seed composition is more affected by geographic regions (∼5%) than by MG (∼2%) (Bandillo et al., 2015), it can be concluded that the effect of lower temperatures on seed composition at higher latitudes might be overcome by the selection of more diverse parental lines in early MGs (Piper and Boote, 1999). Hence, achieving higher protein content without affecting yield or oil content could be obtained by exploring genetic variants of *E* genes.

## Soybean and *E* Genes Allelic Variation

Study of allelic variation and diversity of soybean flowering-time and maturity genes may enhance soybean breeding for particular environments, since achieving appropriate maturity in a target environment maximizes crop yield potential (Langewisch et al., 2017). However, there are not many studies focused on soybean maturity and genes affecting it. This especially stands for European soybean varieties, as most of the studies published so far deal with North-American and Chinese genotypes (Tsubokura et al., 2013, 2014; Jiang et al., 2014; Langewisch et al., 2014, 2017; Zhai et al., 2014; Abugalieva et al., 2016; Valliyodan et al., 2016; Kurasch et al., 2017; Li et al., 2017; Wolfgang and An, 2017)

Soybean collection maintained at Institute of Field and Vegetable Crops Novi Sad, Serbia (IFVCNS), consists of more than 1200 cultivars and lines originating from America (50.1%), Asia (15.6%), and Europe (34.4%). Most of the genotypes belong to MG0 and MGI, but the collection also includes genotypes from MG000 to MGV (Hrustić and Miladinović, 2011). In order to characterize allelic variation and distribution of the major maturity genes (*E1*–*E4*) we conducted the study on 445 genotypes from soybean collections of North-American (NA) ancestral lines (27), Chinese germplasm (14), European varieties (12), genotypes developed and released at IFVCNS - NS varieties (56), NS breeding lines (229), 57 high-protein and 50 high-oil genotypes from NS collection. Our research was mainly focused on identification of the most prominent *E* allelic combinations in NS varieties and breeding lines, with the aim of applying the obtained results for the improvement and targeted breeding of soybean varieties adapted to the environments of Central-Eastern Europe. MGs of the analyzed soybean genotypes ranged from MG000 to MGIII, including several genotypes from NA ancestral population which belong to later MGs MGV-MGVII. All 445 soybean genotypes were characterized using genotyping-by-sequencing approach (Elshire et al., 2011). Sequenced libraries produced a total of 145x10^7^ raw 100-bp DNA reads that were aligned against soybean reference genome Williams 82. Reads aligned in the same genomic region were used for SNP calling, producing initially more than 85,000 SNP markers, where mean sequencing depth per SNP locus was 5. After filtering and application of a combination of criteria, such as minor allele frequency (MAF < 0.05) and the percentage of missing values (PMV < 20%), the obtained marker dataset was used for prediction of the allelic variation of four maturity genes (*E1/e1-as, E2/e2, E3/e3-tr*, and *E4/e4*). Genes *E1* and *E2* possess SNP that gives rise to alleles *e1-as* (G/C; Glyma.W82.a2: 20,207,322 bp) and *e2* (A/T; Glyma.W82.a2: 45,310,781 bp), while *E3* and *E4* genes do not have a functional SNP, causing corresponding occurrence of variant alleles *e3-tr* and *e4* correspondingly. The determinant SNP makers for genes *E1* and *E2* were not present in our dataset, as marker analysis was performed with reduced representation genotyping approach. Thus we examined groups of markers positioned in specific genomic regions which include entire *E* genes (positions given in **Supplementary Table S1**), by characterizing haplotypes aiming to identify the causative haplotype for different alleles, functional, and variant (**Supplementary Table S1**). SNPViz tool was used for haplotype visualization and comparison, in order to provide information on genetic neighborhood in which variable alleles appeared, as described by Langewisch et al. (2014). By comparing the obtained haplotypes with previously published profiles of *E* genes for NA ancestral lines and European varieties (Kurasch et al., 2017; Langewisch et al., 2017), we managed to identify the groups of haplotypes for each allele. Within these regions, lines with known different alleles were distinctively separated to specific haplotypes, which allows assignment of alleles of each gene to specific haplotypes. Moreover, it was made possible to further specify the identified haplotypes and select groups of diagnostic SNP markers able to determine different alleles of *E* genes (**Supplementary Table S1** and **Supplementary Figures S1–S4**). Four SNP markers were identified for *E1* gene which could be distinguished among alleles *E1, e1-as*, and *e1-nl* **Supplementary Figure S1**. Two SNP markers were defined for genes *E2* and *E3*, causing the difference between *E2* and *e2* alleles, as well as between *E3* and *e3-tr* **Supplementary Figures S2, S3**. According to the five identified SNP markers, lines could be classified as *E4* or *e4* allele (**Supplementary Figure S4**). The obtained results were applied for further allele scoring of *E* genes of previously non-genotyped lines, such as NS-varieties and NS-breeding lines. Predictions did not include the non-functional *el-fs* allele, and no distinction could be made between *E3-Ha* and *E3-Mi* alleles, or *e4-oto, e4-tsu, e4-kam*, and *e4-kes*.

**FIGURE 1:**
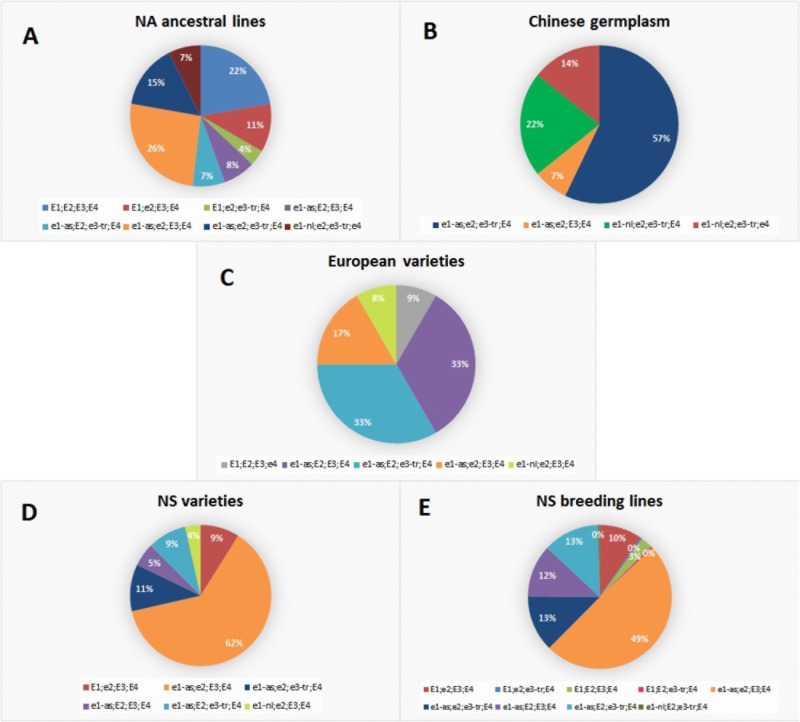
Allelic combinations of maturity loci *E1, E2, E3*, and *E4* over different soybean populations: **(A)** North-American ancestral lines; **(B)** Chinese germplasm; **(C)** European varieties; **(D)** NS varieties; **(E)** NS breeding lines.

All examined genotypes were classified into 12 *E* haplogroups which represent a unique combination of *E1, E2, E3*, and *E4* alleles (**Supplementary Table S2**). Each collection had one major *E* haplogroup which included most of the lines (≥50%) (**Figure 1**). The exception were European varieties with two predominant groups and NA ancestral lines which were distributed almost evenly among several haplogroups. The most abundant NA ancestral haplogroup was *e1-as/e2/E3/E4* with 26% of genotypes. Over 20% of genotypes had *E1/E2/E3/E4*, which coincides with the high number of late-maturing genotypes included in the NA collection. This is concordant with molecular maturity model proposed by Langewisch et al. (2017) where early-maturing lines had non-functional *e1/e2/e3-tr* and late-maturing lines had *E1/E2/E3*. None of the lines from the Chinese collection carried functional photoperiod-sensitive *E1* allele, while two photoperiod-insensitive alleles (*e1-nl* and *e1-as*) were identified at *E1* locus. Contrary to previous reports, where most of the tested accessions of breeding collections from China were *E1/e2/E3/E4* followed by *e1-as/e2/e3/E4* (Jia et al., 2014; Jiang et al., 2014), the most abundant haplogroup in Chinese germplasm in our collection was *e1-as/e2/e3-tr/E4* including 57% of lines. The remaining lines mostly contained *e1-nl* allele with *e1-nl/e2/e3-tr/E4* and *e1-nl/e2/e3-tr/e4*, covering 21 and 14% of genotypes, respectively. Of all examined genotypes, only three lines from the NA ancestral and Chinese germplasm had the non-functional *e4* allele, which is in agreement with the fact that *e4* alleles were found in small geographical locations with high latitudes and low temperature (Tsubokura et al., 2013; Langewisch et al., 2014). European varieties from our collection differed from the varieties studied by Kurasch et al. (2017) in their *E* haplotype, as more than 30% of varieties from our collection were *e1-as/E2/E3/E4* and the same number of genotypes had *e1-as/E2/e3-tr/E4*, compared to 1.3 and 12%, respectively, in the study of Kurasch et al. (2017). The haplogroup *e1-as/e2/E3/E4* comprised 17% of lines in European varieties. Similar to the results of Kurasch et al. (2017), NS varieties primarily had *e1-as/e2/E3/E4* haplotype identified in 63% of genotypes. The same haplotype was found to be predominant in the Kazakh breeding collection (Abugalieva et al., 2016), which was attributed to the frequent exchange of germplasm with partners from former USSR countries. The second largest haplotype in NS varieties is *e1-as/e2/e3-tr/E4* with 11% of genotypes. All other allelic combinations were found in less than 10% of genotypes. For the *E1* locus, allele *el-as* was detected in 88% of NS varieties classified into MG000-MGIII. Nearly half of the NS breeding lines were predicted to be *e1-as/e2/E3/E4*, with the additional 13% of genotypes predicted as *e1-as/e2/e3-tr/E4* and 13% of *e1-as/E2/e3-tr/E4*.

Novi Sad collection exhibited a high diversity of allelic combinations of *E* genes. The most abundant haplogroup in NS varieties and breeding lines was *e1-as/e2/E3/E4*. The same haplogroup comprised 26% of genotypes in NA ancestral lines which represent more than 85% of the pedigree of NS varieties, indicating that *e1-as/e2/E3/E4* haplotype was favored by artificial selection during soybean breeding under the environmental conditions of Central-Eastern Europe. NS varieties and breeding lines had predominantly *e1-as* allele, indicating that they might have been purposely selected for similar maturity and high-yield capacity. This finding is compatible with identified genomic regions affected by positive selection during breeding of NS soybean varieties, where two loci (*Satt357* and *Satt557*) on chromosome 6 were identified as strong positive selection candidates (Tomičić et al., 2015). Similar results were obtained during evaluation of the allelic variants at the *E* genes in 75 European cultivars from five MGs (MG000-MGII), where none of the cultivars carried functional *E1* allele (Kurasch et al., 2017). It was concluded that the *E1* locus plays a major role in early flowering and maturity, and that photoperiod insensitivity at this locus is a probable requirement for soybeans adapted to Central or Northern Europe.

**FIGURE 2:**
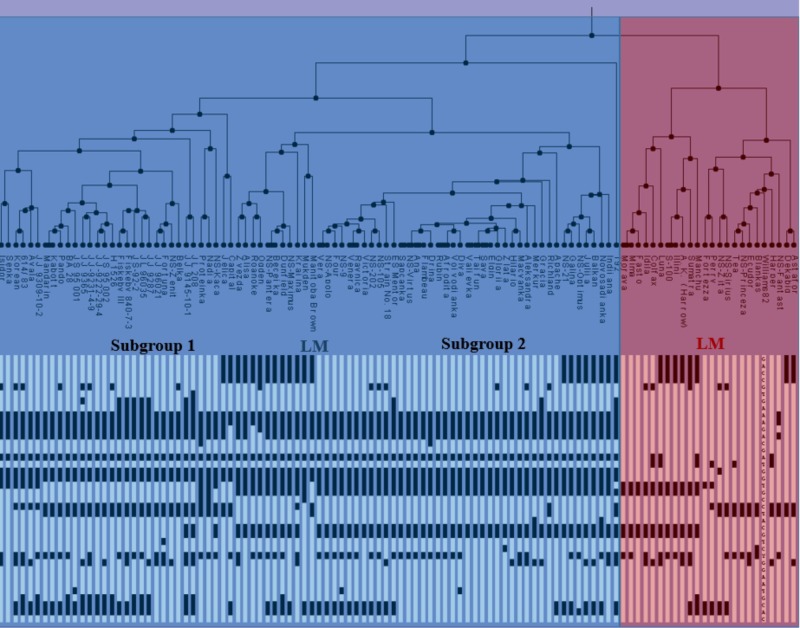
Clustering tree and haplotypes of NA ancestral lines, Chinese germplasm, European varieties, and NS varieties, based on the diagnostic SNP markers for four analyzed *E* genes, (EM, early maturing; LM, late maturing).

Based on the used SNP markers, two major clusters can be identified and described as early material (EM) consisting of MG000-MGI and late material (LM) consisting mainly of MGII-MGIV genotypes (**Figure 2**). The first subgroup from EM included Chinese germplasm, NA ancestral lines and NS varieties. Recessive haplotype *e1-as/e2/e3-tr/E4* was the dominant haplotype in this subgroup. The majority of NS varieties belonged to the second subgroup, along with a few NA and European varieties. The most abundant allelic combination in this subcluster was *e1-as/e2/E3/E4*, which represents the major haplogroup within environmental conditions of Central-Eastern Europe. The second group mostly comprised LM genotypes, including NA ancestral lines, European and NS varieties, from MGI to MGIV, with the exception of NS Princeza which belongs to MG0. Most lines from the LM cluster had three or four dominant alleles. The observed grouping of genotypes into late and early maturing clusters indicates that alleles of *E1*–*E4* genes can be used in the assessment and classification of the assortment according to maturity. However, four genes used in this study are not sufficient to make the precise distinction between different MGs, as there is no unique allelic combination specific for each group. Further work is needed to identify molecular mechanisms behind soybean photoperiod sensitivity, and linking specific allelic combination with a certain MG.

## Future Prospects

Flowering time and maturity are agronomically important traits, which affect soybean adaptation, quality traits, and yield. Complete understanding of their regulation will therefore allow breeding of varieties with optimal flowering or maturity for particular geographic regions. Considering this, we have studied the distribution of different haplogroups of *E* genes in soybean collections of NA ancestral lines, Chinese germplasm, European varieties, NS varieties, and NS breeding lines. Overall, the observed allelic combinations of *E1*–*E4* genes significantly determined the adaptation of varieties to different geographic regions, although they have different impacts on maturity.

The study was also the first attempt to assess NS soybean varieties and breeding line adaptation based on *E* gene variation, as well as comparatively assess *E* gene variation in NA, Chinese and European genotypes. The study aims to identify the most prominent *E* allelic combinations in NS genotypes and apply the obtained results in target breeding and introgression of yield and quality traits for environments of Central-Eastern Europe. As *e1-as/e2/E3/E4* was the most common haplotype in the NS population comprising high-yielding varieties grown throughout Central and Eastern Europe, the specific allele combination was proposed as the optimal combination for this environment.

The obtained data provide useful information about the selection of parental genotypes most appropriate for use in local breeding programs for improved soybean productivity. This allows plant breeders to transfer traits more effectively into different MGs, enhance germplasm exploitation, and increase overall efficiency of soybean breeding. Furthermore, the obtained knowledge will significantly contribute to the marker-assisted selection and molecular breeding of soybean, and increase breeding efficiency by enhancing genomic prediction models.

## Author Contributions

JM, MĆ, SB-T, and VuĐ planned and designed the experiment. MĆ, KP, VuĐ, and VoĐ performed the research. JM, DM, MĆ, and VuĐ contributed to the interpretation and analysis of results. JM, MĆ, and DM wrote and approved the manuscript.

## Conflict of Interest Statement

The authors declare that the research was conducted in the absence of any commercial or financial relationships that could be construed as a potential conflict of interest.

## Supplementary Material

FIGURE S1 Diagnostic haplotypes of NA ancestral lines, Chinese germplasm, European varieties and NS varieties, based on selected SNP markers for *E1* gene.

FIGURE S2 Diagnostic haplotypes of NA ancestral lines, Chinese germplasm, European varieties and NS varieties, based on selected SNP markers for *E2* gene.

FIGURE S3 Diagnostic haplotypes of NA ancestral lines, Chinese germplasm, European varieties and NS varieties, based on selected SNP markers for *E3* gene.

FIGURE S4 Diagnostic haplotypes of NA ancestral lines, Chinese germplasm, European varieties and NS varieties, based on selected SNP markers for *E4* gene.

TABLE S1 SNP calls located in the genomic regions of *E1*, *E2*, *E3*, and *E4* genes in soybean collections of North-American (NA) ancestral lines, Chinese germplasm, European varieties, and genotypes developed and released at Institute of Field and Vegetable Crops – NS (Novi Sad) varieties, NS breeding lines, high-protein and high-oil genotypes from NS collection.

TABLE S2 Allelic variation on the *E1*, *E2*, *E3*, and *E4* maturity genes in soybean collections of North-American (NA) ancestral lines, Chinese germplasm, European varieties, and genotypes developed and released at Institute of Field and Vegetable Crops – NS (Novi Sad) varieties, NS breeding lines, high-protein and high-oil genotypes from NS collection.
