# Supplementary figures and images for: Allelic Variation and Distribution of the Major Maturity Genes in Different Soybean Collections
Source: Front Plant Sci. 2018 Sep 4;9:1286. doi: 10.3389/fpls.2018.01286 (PMC6131654; doi:10.3389/fpls.2018.01286)

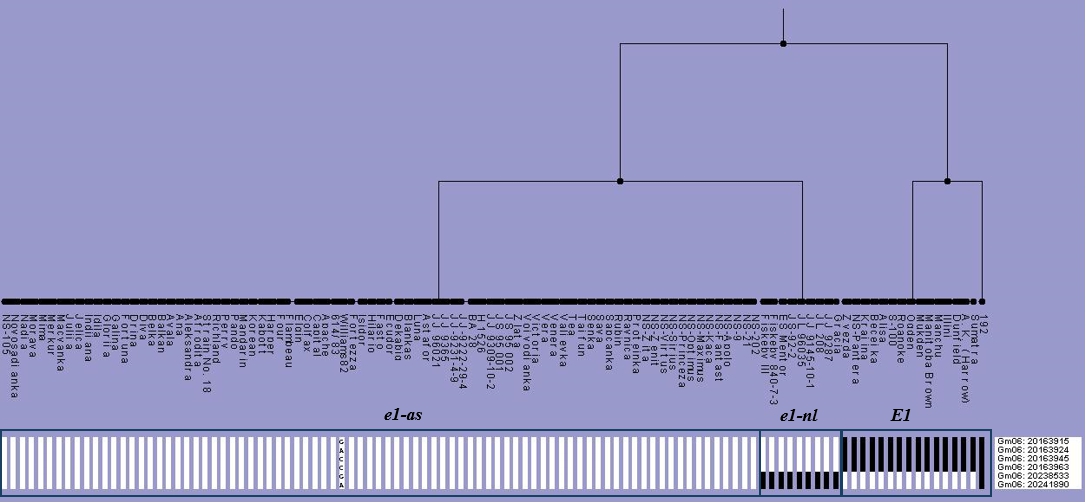

Supplement: FIGURE S1 — Diagnostic haplotypes of NA ancestral lines, Chinese germplasm, European varieties and NS varieties, based on selected SNP markers for E1 gene. [file Image_1.JPEG]

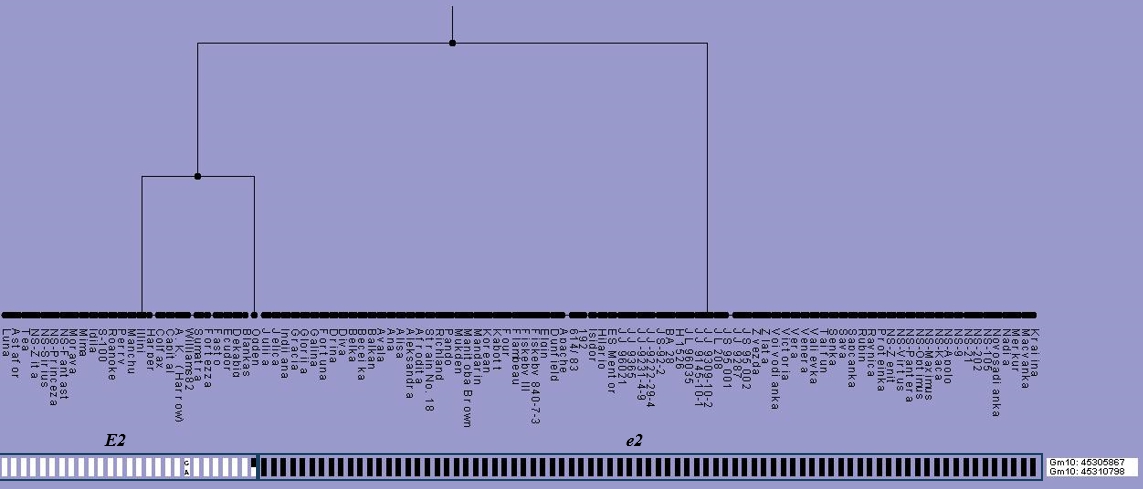

Supplement: FIGURE S2 — Diagnostic haplotypes of NA ancestral lines, Chinese germplasm, European varieties and NS varieties, based on selected SNP markers for E2 gene. [file Image_2.JPEG]

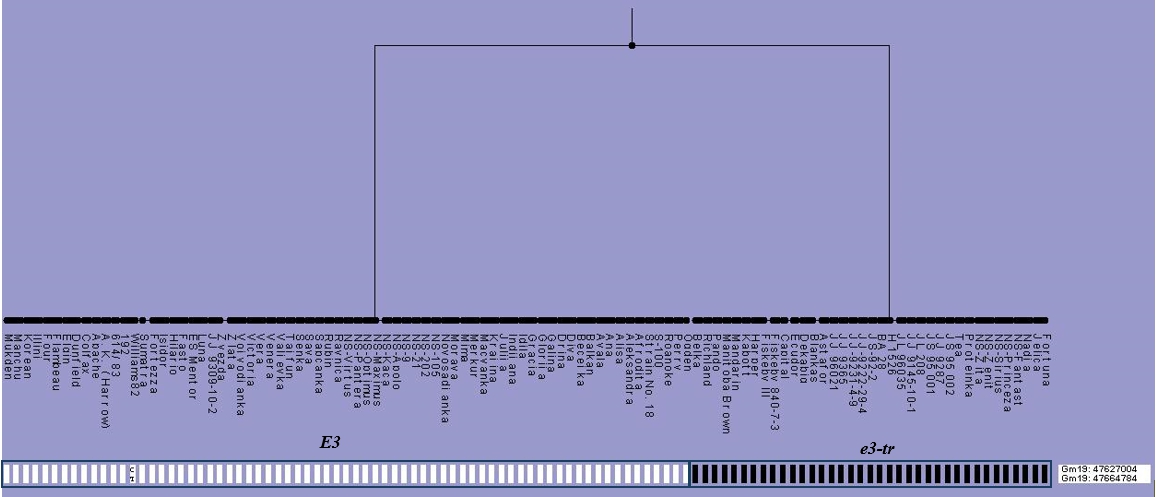

Supplement: FIGURE S3 — Diagnostic haplotypes of NA ancestral lines, Chinese germplasm, European varieties and NS varieties, based on selected SNP markers for E3 gene. [file Image_3.JPEG]

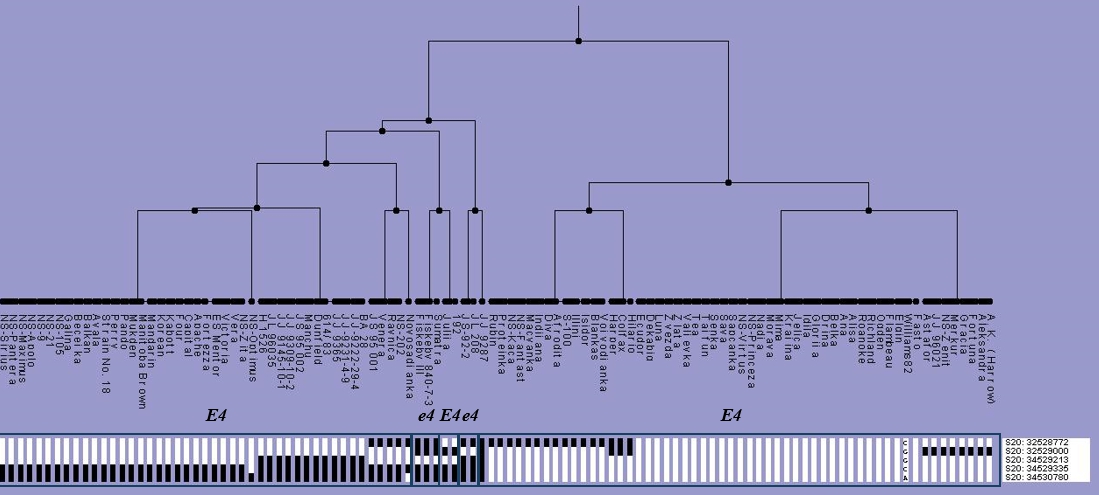

Supplement: FIGURE S4 — Diagnostic haplotypes of NA ancestral lines, Chinese germplasm, European varieties and NS varieties, based on selected SNP markers for E4 gene. [file Image_4.JPEG]
